# Improving mental health response in earthquake-prone regions: recommendations following the recent earthquake in Türkiye

**DOI:** 10.1192/bji.2024.23

**Published:** 2024-11

**Authors:** Rodrigo Ramalho, Rojda Ersönmez, İdil Kına, İrem Keçeci, Ramin Apparao, Eugene B. Y. Koh, Bülent Coskun

**Affiliations:** 1 Department of Social and Community Health, School of Population Health, University of Auckland, Auckland, New Zealand. Email: r.ramalho@auckland.ac.nz; 2 Department of Emergency Medicine, Besni State Hospital, Adiyaman, Türkiye; 3 Department of Psychiatry, Yale School of Medicine, New Haven, Connecticut, USA; 4 Department of Psychiatry, Uskudar University, Istanbul, Türkiye; 5 Faculty of Medicine and Health Sciences, Universiti Putra Malaysia, Serdang, Malaysia; 6 Association for Community Mental Health Promotion, Kocaeli, Türkiye

**Keywords:** Natural disasters, earthquakes, mental health, public health, health planning

## Abstract

We are members of Students’ Education, Empowerment and Development in Mental Health, a group of medical students and psychiatrists from around the globe under the Psychiatry, Medicine and Primary Care Section of the World Psychiatric Association. In this article, we put forward recommendations to help improve the mental health response in disaster-prone regions such as Türkiye. We recommend a three-step multi-tiered mental health response system that could significantly help to address the immediate, short-term and long-term mental health needs of communities directly affected by a disaster. The recommendation draws from the relevant literature and, most importantly, from our lived experiences of living in earthquake-prone countries.

The recent earthquake in the Republic of Türkiye in February 2023 was a catastrophic disaster. As highlighted by Makwana,^1^ preparedness and community empowerment can help those directly affected by disasters, and the people responsible for disaster preparations in Türkiye have expressed their willingness to work on these aspects.^2^ Some recommendations have been put forward,^3^ but a more systematic response emphasising preparedness and community empowerment is needed.

We are members of Students’ Education, Empowerment and Development (SEED) in Mental Health. This is a group of medical students and psychiatrists from around the globe under the Psychiatry, Medicine and Primary Care Section of the World Psychiatric Association. The SEED group was founded as a project group working in collaboration with a non-governmental organisation from Türkiye, Association for Community Mental Health Promotion (ACMHP). This article draws on a non-systematic literature review and our ongoing monthly Intergenerational Meetings with internationally renowned experts, such as Professors Norman Sartorius, Rachel Jenkins, Afzal Javed and David Baron; conversations were recorded and are shared on the ACMHP YouTube channel (https://www.youtube.com/@trsgd/streams). It also, most importantly, draws on our lived experiences of living in earthquake-prone countries, including Türkiye. R.E., a general practitioner in Adiyaman at the time, confronted the direct consequences of inadequate disaster preparedness and coordination during the recent earthquake, whereas I. Kına., a psychiatry resident in Istanbul at the time, witnessed the compounded trauma of survivors who had lost their support systems. I. Keçeci, whose family had to relocate with her owing to the disaster, experienced first-hand the need for comprehensive mental health training for all medical professionals, a situation echoed by B.C.'s witnessing of persistent coordination failures since the 1999 Marmara earthquake; B.C. also highlighted the importance of developing a plan that includes further preparedness for and responsiveness to future disaster situations, including a to-be-expected earthquake in Istanbul.

We recommend a three-step, multi-tiered response system that we believe could prove pivotal in addressing the immediate, short-term and long-term mental health needs of communities affected by an earthquake. Preparation is crucial. In the preparation step, the goal should be to develop a well-coordinated and sustainable response system that includes immediate and mid-to-long-term responses. As with the recent earthquake, immediate responses are likely to be led by members of the local community. The response system should enable and guide local healthcare providers to support their community. Countries should develop a national network of healthcare professionals that can be promptly mobilised in response to a disaster.^4^ In addition, everyone involved in the response should receive psychological first aid (PFA) training. Appropriate PFA training can promote resilience and enhance self-efficacy in supporting people,^5^ and it will provide healthcare providers and volunteers with the necessary tools to deliver prompt care in the acute phase. Healthcare professionals should receive this training early in their careers, allowing trainees and established professionals to support the directly affected community. The World Health Organization also offers a five-session stress management course for large groups, called Self-Help Plus (SH+), which is available in Turkish.^6^ Specialists and non-specialists who are part of the response system could also access this training. In addition, the Mental Health Gap Action Programme,^7^ another resource provided by the WHO, can help enhance primary care providers’ capacity to detect and manage mental health conditions in humanitarian settings.

The second step of the system is triggered when disaster strikes. During this time, local primary care professionals and other front-line workers will be best positioned to provide prompt mental health support to the community. Those who have received SH+ training could also provide the general population with additional support, and others may be encouraged to take this training. Mental health professionals, in person if they are locals or via telemental healthcare if not, could provide additional support when someone is identified as experiencing moderate to severe mental health disorders. When necessary, psychiatrists – or other prescribing professionals – could further assist those with complex needs. This structure mirrors the three-tier system proposed by Pandya and colleagues^8^ in India during the COVID pandemic, requiring the least specialist care at the first tier and increasing the country's capacity to support the community without significantly affecting the larger health system. It is also important to allow a quick turnover of healthcare providers to avoid vicarious trauma and burnout among these professionals, and healthcare providers should also start receiving mental health support as soon as possible.

All responses during the disaster will require careful planning, preparation, political support and funding. As emphasised by the Inter-Agency Standing Committee,^9^ basic services and security are a key layer of intervention when providing mental health and psychosocial support in emergency settings. Moreover, providing basic needs such as food, shelter and safety should come first in the response. Thus, preliminary preparations are necessary to ensure that all basic needs of the directly affected community are met; these, of course, should also be secured in the long term. The local and wider national community should have clear pathways to channel their support, e.g. collecting and distributing food, clean water and any other needed supplies. These preparations should require low resources to be deployed. The system should also deploy recovery and relocation efforts as soon as it is safe to do so. Healthcare professionals travelling to support the community should be offered transport, accommodation and meal arrangements. Likewise, mental healthcare providers supporting local primary care professionals via telehealth should be offered protected time and the necessary resources. Multi-sectoral collaboration and partnerships will be required to offer healthcare professionals and the general population the resources they need to support those directly affected by the disaster. It is also important to provide the local, national and international communities with clear and easily accessible information about priority needs and planned actions to allow everyone to join in the efforts.

As a third and final step, the response system should also account for the extended impact of the disaster, including long-term mental health effects. In the post-event phase, the system should continue to include relief-centred management goals but also start showing more explicit signs of health promotion, recovery and rehabilitation-focused strategies. The system should account for sustained efforts for early detection and care of people presenting with clinical and subclinical mental health disorders, support for their families, and promotion of resiliency and flourishing within the community. At this stage, community-based interventions are crucial. Local community members should be allowed to process the psychological impact and social and structural disruption caused by the disaster as soon as possible, as well as contributing to relief and rebuilding efforts. Culturally responsive interventions that empower the community, foster their resiliency and build their collective self-efficacy are pivotal in the healing process. The system should also include periodic monitoring, evaluation and adjustment plans at all stages to ensure it remains responsive to the community's changing needs.^10^ At this point, we also acknowledge that disaster prevention is just as crucial, if not more so, for community mental health, and further work with this focus should be pursued, as has been suggested by the World Health Organization.^11^

The recent earthquake in the Republic of Türkiye brings to mind the Voltaire–Rousseau debate during the 1755 Portugal earthquake. Like Rousseau, we believe that rather than viewing disaster as part of destiny's plan, preparedness can help significantly reduce the impact of a disaster. But if we can put forward a well-structured response system, we might be able to efficiently prevent and significantly address some of the consequences. We hope the recommendations in this article, summarised below in Table 1, represent a useful starting point.

**Table 1:** Mental health response framework for disaster-prone countries

Steps^a^	Recommended responses
Step 1: prevention	As suggested by the World Health Organization in 2024, an emergency cycle must include prevention as a key first step, followed by preparation, response and recovery, steps intimately aligned with those presented below.
Step 2: coordinated preparedness	Developing a national network of healthcare professionals volunteering to support communities affected by a disaster. Providing psychological first aid training to everyone providing support. Healthcare professionals should receive this training early in their career pathways. Additional training could include Self-Help Plus (for specialists and non-specialists) and the Mental Health Gap Action Programme (for primary care providers). Multi-sectoral collaboration and partnerships are necessary to ensure the resources required to assist those directly affected during a disaster. They should be part of or support the abovementioned network. Developing or strengthening pathways that enable and guide local healthcare providers to support their community during a disaster. An easy-to-access and understand platform could be developed to gather and disseminate information.
Step 3: immediate response	Provide clear and easily accessible information about priority needs and planned actions. Communicate clear pathways to channel the support of the local, national, and international communities towards those most vulnerable. Ensure that the basic needs of the directly affected community are covered. As soon as it is safe to do so, the system should also deploy recovery and relocation efforts. Local primary care professionals and other front-line workers are to provide prompt mental health support to the community. When someone is identified as experiencing moderate to severe mental health disorders, mental health professionals should provide additional support, either in person or via telemental healthcare. People experiencing more complex needs are to be supported by psychiatrists, either in person or via telemental healthcare. Allow a quick turnover of healthcare providers to avoid vicarious trauma and burnout. Healthcare providers should receive mental health support as soon as possible. Healthcare professionals travelling to the community or providing additional assistance via telemental health should be supported to do so.
Step 4: sustained healing	Ensure all basic needs of the directly affected community are met in the long term. Continue relief-centred management goals. Include health promotion, recovery and rehabilitation-focused strategies. Continue implementing steps for early detection and care of people presenting with clinical and subclinical mental health disorders. Provide support to the families of those most directly affected by the disaster. Culturally safe and community-based interventions promoting resiliency and flourishing within the community should be implemented as soon as possible. Consultation and collaboration with the community during relief and rebuilding efforts are key. The system should include periodic monitoring, evaluation, and adjustment plans at all stages.

a. Some activities are not restricted to a single step, such as coordination, consultation and monitoring.
